# On a Loophole in Causal Closure

**DOI:** 10.1007/s11406-016-9791-y

**Published:** 2017-02-15

**Authors:** Johan Gamper

**Affiliations:** Department of Philosophy, School of Culture and Education, Södertörn University, Alfred Nobels allé 7, 141 89 Huddinge, Sweden

**Keywords:** Causal closure, Philosophy of mind, Philosophy of physics

## Abstract

Standard definitions of causal closure focus on where the causes in question are. In this paper, the focus is changed to where they are *not*. Causal closure is linked to the principle that no cause of *another* universe causes an event in a particular universe. This view permits the one universe to be affected by the other via an *interface*. An interface between universes can be seen as a domain that violates the suggested account of causal closure, suggesting a view in which universes are causally closed whereas interfaces are not. On this basis, universes are not affected by other universes directly but rather indirectly.

## Introduction

Let us say that a *universe* is a domain of things of a certain ontological status, e.g., *physical* things or *mental* things. Causal closure, then, concerns the possibility that there may be more than one universe. In this paper, we in principle go back to Descartes and Princess Elisabeth and their discussion of the relation between substances of mind and body. We implicitly address the question of whether there are non-physical objects and therefore one or more domains of non-physical objects. Are there, e.g., purely *mental objects* or *mathematical objects*? Explicitly, however, we address the question of whether we can save causal closure without giving up on the possibility of pluralism.

The structure of the argument is as follows: Causal closure seems to cause serious problems. When we consider causal closure of the physical domain, one problem is that it does not seem to reach the cause of a very first physical event. Additionally, this problem affects any universe. If there is a domain of mental objects and causal closure is true, then it seems that we are unable to find the cause of a first mental object. What may be worse is that causal closure prevents us from coming into contact with other potential universes. If there are mental objects, whereas ‘we’ are physical, then any contact seems impossible. Additionally, a consistent argument for our inhabiting a physical domain is as strong as a consistent argument for our inhabiting any non-physical domain. Simultaneously, however, the soundness of causal closure seems analytical: The domain of, e.g., the physical on a conceptual level should contain the things that any physical thing causes and, vice versa, all things that cause any physical thing. The suggested loophole is found by disentangling from this ‘analytical’ truth and then seeking an alternative without letting go of what seems necessary with causal closure.

Therefore, the ‘universes’ that are discussed have ‘substances’ or contents, but any two universes have contents that cannot causally interact. For instance, you cannot move the things on your desk by thought. The question that is addressed concerns the possibility that, nevertheless, there is some type of causal connection between some two different universes. The key, actually, will be found in an alternative way of stating the first sentence in this very paragraph: ‘Therefore, the “universes” that are discussed have “substances” or contents, but any two universes have contents that cannot causally interact’.

Above, we assert that a universe is a domain of things of a certain ontological status. Without stating any existential claims, we can exemplify this point with, first, ‘a domain of physical things’, D1, and, second, ‘a domain of mental things’, D2. The first sentence in the previous paragraph, then, claims that no set of things in D1 has any causal bearing on any set of things in D2 (and vice versa). The key now is to take a step back and suggest that it is sufficient for causal closure that D1 and D2 have no causal interaction, leaving it open as to there being any ‘causal bearing’ between the two. Naturally, this concept appears paradoxical, but it is our starting point towards the ‘loophole’ that arises when we change perspective from where the causes are to where they are not. According to a standard view of causal closure, the causes of the things in the domain of the physical are in that very domain. If we alter this way of understanding causal closure and examine the alternative that the causes of the things in the domain of the physical are not in any other domain (of some non-physical things), then the loophole emerges if there are interfaces between domains of things that themselves are not domains of things.

Next, we focus on a standard definition of causal closure.

## Causal Closure 1.0

One way of stating the principle of physical causal closure is this: If you pick any physical event and trace out its causal ancestry or posterity, that will never take you outside the physical domain. That is, no causal chain will ever cross the boundary between the physical and the nonphysical. (Kim 1998, p. 40).

Kim has elaborated on this principle, arguing that the mind seems physical. To push it a step further, if we assume that we are physical (to any degree), then everything that we causally interact with is physical. Although this principle is common in many camps, it has an odd feature. First, if we generalize the principle, i.e., *any* domain is causally closed, then it is neutral as to what we are and everything around us is. For instance, if we, to any degree, are mental, then everything we causally interact with is mental. Additionally, if something we causally interact with is mathematical, then everything we causally interact with ends up being mathematical.

Actually, Tegmark (2008) has given an account that takes our physical world to be mathematical: ‘I argue that with a sufficiently broad definition of mathematics, it [the *External Reality Hypothesis*] implies the *Mathematical Universe Hypothesis* […] that our physical world is an abstract mathematical structure’ (p. 101). The External Reality Hypothesis is the common sense view that ‘*[t] here exists an external physical reality completely independent of us humans*’ (p. 102). Tegmark shows that this hypothesis implies the Mathematical Universe Hypothesis, which is the view that ‘*[o] ur external physical reality is a mathematical structure*’ (p. 102) and ‘[a] *mathematical structure* is … *abstract entities with relations between them*’ (p. 104):

the conventional approach holds that a theory of mathematical physics can be broken down into (i) a mathematical structure, (ii) an empirical domain and (iii) a set of correspondence rules which link parts of the mathematical structure with parts of the empirical domain. If the [Mathematical Universe Hypothesis] is correct, then (ii) and (iii) are redundant in the sense that they can, at least in principle, be derived from (i). (Tegmark 2008, p.140).

The eventuality that our world is mathematical is also noted by Dorr (2010):

Perhaps we cannot even rule out a priori the even more radical hypothesis that some or all of the objects of our acquaintance are *themselves* mathematical objects, as in the ‘hyper-Pythagorean’ views entertained by Quine (1976) and Tegmark (2008). (p. 141).

Therefore, Kim’s generalized principle does not permit us to know where (in what type of universe) we are. Additionally, if the principle is true, then there may be other types of existences, but we can never come into contact with them. The lesson is that the principle is not that informative. In the next section, we specify a causal closure definition and attempt to alter it to improve on the situation.

## Causal Closure 1.1

For Steinhart (2009), ‘[a] universe is *causally closed* iff all causes of events in the universe are in the universe, and all effects of events in the universe are in the universe’ (p. 43). This definition permits events to be cause-less. To improve on Steinhart’s definition, we can consider causal closure from the perspective of a potential multitude of universes. The principle of causal closure, evidently, is about *one* universe, not about there being *only* one universe. From this perspective, it seems sufficient to focus on the first condition of Steinhart’s definition. If we alter ‘*the* universe’ to ‘*a/the same* universe’, then we obtain ‘all causes of events in a universe are in the *same* universe’. Now, it seems superfluous to add the second, modified condition that ‘all effects of events in a universe are in the *same* universe’ since the causes of events do not leave the particular universe. If ‘an effect of an event in a universe were in *another* universe’, then that would contradict the principle that ‘all causes of events in a universe are in the *same* universe’ since the event in this case is clearly a cause.

Therefore, on the multiverse view, we can define causal closure as ‘all causes of events in a universe are in the *same* universe’. To improve on this definition, we will actually make it *less* strong. First, we conclude that if there is only one universe, then we do not need to concern ourselves with closure principles. Next, we focus on whether, if there is more than one universe, we can state the weaker principle that no cause of *another* universe causes an event in our universe. On this view, if there is only one universe, then we are secure as to where the causes come from; additionally, if there is more than one universe, then we are safe from having causes that come in from any of the other universes.

## A Loophole

Another odd feature of both Kim’s principle and Steinhart’s definition is that on those bases, a very first thing or event in a universe cannot be caused. If all causes of events are in the universe, then the cause of the first event is also in the universe but that is impossible, given that this event did not cause itself (if we take it that causes are prior to their effects). If we adopt the updated definition of causal closure, however, then we have potential access to a ‘causal closure loophole’. With the weaker claim that no cause of *another* universe causes anything in our universe, we gain the possibility of an *interface* between universes.

If all causes of events in a universe belong to either the universe itself *or* an interface between the universe itself and *another* universe, then the principle that no cause of *another* universe causes an event in our universe is not violated. For instance, if we view the frequently conjectured event of the Big Bang as an interface between universes, then with the altered definition, we are allowed to view the universe as causally closed, even though the cause of the Big Bang is external to the universe because the Big Bang is not part of our universe. Thus, on this view, causal closure permits the one universe to be affected by the other via an interface. On this basis, universes are not affected by other universes directly but rather indirectly.

To obtain a clear view of this picture, we recapitulate our discussion. Steinhart’s (2009) standard definition of causal closure tells us that ‘[a] universe is *causally closed* iff all causes of events in the universe are in the universe, and all effects of events in the universe are in the universe’ (p. 43). On the one hand, this definition leaves a very first event in a universe cause-less unless it causes itself (or has its cause preceding it). On the other hand, this definition permits cause-less events, which seems to have the effect that we can and must abandon any inquiries into a first event of a (our) universe. However, if we alter this definition, focusing on where the causes are *not* instead of where they are and state that a universe is causally closed if and only if no cause of *another* universe causes an event in our universe, then we are free to look closer to the possibility that a first event in a universe has a cause.

Naturally, this cause of a first event of a universe cannot belong to another universe. However, on the ‘loophole’ view of causal closure, there is another possibility. We can add a disjunct to the phrase ‘all causes of events in a universe belong to the very universe’ and state that ‘all causes of events in a universe belong to the universe itself or to an interface between the universe itself and *another* universe’ without violating the updated version of causal closure.

## Interfaces between Universes

An interface between universes, first, cannot be a universe. This assertion calls for investigations into what a universe is and what a universe is not, work that must be postponed. Second, we nevertheless have some facts to consider. On the ‘loophole’ view, universes have ‘events’, ‘causes’ and ‘effects’. For instance, D1 and D2 above have these occurrences. When we examine an interface, however, it appears as though we have the same ingredients (why the ‘loophole’ view perhaps could end up being incoherent^1^): If the cause of an (first) event in our universe comes from an interface, then that interface contains a cause. Additionally, if we were to utilize the updated definition, it should be possible to consider a cause of an event in the interface, supposedly implying the existence of an ‘effect’ in the interface. If the cause, the effect or both constitute an event, then we have the triad of ‘event’, ‘cause’ and ‘effect’ in the interface.

That we have the same elements in a ‘universe’ and in an ‘interface’ does not necessarily imply that the ‘loophole’ is closed. A resolution can be conceived in terms of, e.g., quantity. A mathematical point, e.g., consists of the kind ‘points’ just as the mathematical line does. Nevertheless, the mathematical line is not a point and vice versa.^2^

Another alternative for the possibility of interfaces of the kind discussed is that universes have dimensional properties that are not shared with interfaces. An obvious possible scenario is that an interface is not causally closed. If we say that a universe is causally closed and that an interface is not, then we immediately observe that an interface is not a universe. Additionally, if something in the interface has its cause outside it, it is true that the interface is not a universe. Additionally, we can let the updated definition of causal closure come to work and observe how it makes sense that the cause of something in the interface comes from somewhere else. With the updated version of causal closure, it is not necessary that the effects of causes in a universe take place in that universe: Provided that we move with universes, the effects of causes in a universe stay in that very universe since any other universe has no effects with causes coming from it. If we move with a mixture of universes and an interface, however, then it is fully appropriate to consider that an event in a universe causes an effect in an interface. An effect in a universe with its cause coming from an interface is also permitted on the loophole view, as noted above in regard to the Big Bang.

Thus, it is not necessary that interfaces be universes. However, whether there are any interfaces at all, is another story.

## Footnotes

1 It would be incoherent if it stated that 1. there is at least one interface between universes and 2. the stating of an interface between universes is inconsistent. On the contrary, the ‘loophole’ view (implicitly) states only that causal closure is consistent with the possibility of an interface. If an interface, per se, is impossible, then we should drop the second disjunct in the updated definition of causal closure (‘all causes of events in a universe belong to the universe itself or to an interface between the universe itself and *another* universe’).

2 For an analogy in this vein, regard an incidence axiom of Hilbert: ‘There exist at least three points that do not lie on the same line’. Concerning a universe, we could postulate that it contains at least *x* (x > 1), e.g., ‘causes’, whereas an interface could be postulated to contain, say, *one* ‘cause’.

## References

[CR1] Dorr, C. (2010). *Of Numbers and Electrons*. *Proceedings of the Aristotelian Society* 110 (2 pt2):133–181.

[CR2] Kim, J. (1998). *Mind in a Physical World: An Essay on the Mind-Body Problem and Mental Causation*. MIT Press.

[CR3] Quine, W. van. Orman 1976: ‘Whither Physical Objects?’. In R. S. Cohen, Paul K. Feyerabend, and Marx W. Wartofsky (eds.), Essays in Memory of Imre Lakatos, pp. 497–504. Dordrecht: D. Reidel.

[CR4] Steinhart, E. (2009). *More Precisely: The Math You Need to do Philosophy*. Broadview Press.

[CR5] Tegmark M (2008). The mathematical universe. Foundations of Physics.

